# Recent Advances in Bioinspired Cu-Directed C–H Hydroxylation Reactions

**DOI:** 10.1021/acs.accounts.5c00476

**Published:** 2025-08-27

**Authors:** Sunipa Goswami, Isaac Garcia-Bosch

**Affiliations:** Department of Chemistry, 6612 Carnegie Mellon University, Pittsburgh, Pennsylvania 15213, United States

## Abstract

Cu-dependent metalloenzymes catalyze a wide array of oxidative transformations using O_2_ as an oxidant under mild conditions. These include the hydroxylation of challenging organic substrates (e.g., oxidation of methane to methanol in particulate methane monooxygenase) and the regio- and enantioselective hydroxylation of complex molecules (e.g., benzylic hydroxylation of dopamine to noradrenaline in dopamine-β-monooxygenase). Lytic polysaccharide monooxygenase enzymes (LPMOs) promote the C–H hydroxylation and subsequent cleavage of the polysaccharide chains found in natural materials such as cellulose or chitin. Recent reports on the reactivity of LPMOs suggest that, instead of O_2_, these Cu-dependent metalloenzymes utilize H_2_O_2_ as an oxidant. In 2015, our research lab reported that the catalytic hydroxylation of strong C–H bonds (e.g., cyclohexane) using Cu and H_2_O_2_ proceeded via formation of nonselective Fenton-like oxidants (hydroxyl and hydroperoxyl radicals). To achieve regioselectivity, LPMOs bind the organic substrate before exposing the Cu center to the oxidant, a reaction that leads to the formation of a highly organized ternary complex prior to substrate hydroxylation (i.e., metal–substrate–oxidant adduct). Based on this concept, our research lab has pioneered the use of Cu, directing groups, and green oxidants to promote the site-selective hydroxylation of ketones and aldehydes. In our first report on this topic, we carried out an extensive mechanistic analysis on the Cu-directed sp^3^ C–H hydroxylation reactions developed by Schönecker and co-workers. Our findings suggested that the reaction between Cu^I^ and O_2_ did not lead to the formation of dinuclear Cu_2_O_2_ (as it was previously suggested) but produced Cu^II^ and H_2_O_2_, which generated mononuclear Cu^II^-hydroperoxide oxidants. Based on our mechanistic analysis, we redesigned the reaction conditions to utilize Cu^II^ and H_2_O_2_, which improved the yield, cost, and practicability of the Schönecker oxidations. Since then, our research lab has broadened the scope of substrates that can be oxidized using Cu, H_2_O_2_, and bidentate directing groups to include the γ-hydroxylation of sp^2^ C–H bonds and β-hydroxylation of sp^3^ C–H bonds. Our latest reports have focused on the regioselective hydroxylation of substituted unsymmetrical benzophenones (which occurred via the formation of an electrophilic CuOOH species) and, for the first time, enantioselective C–H hydroxylation reactions via the formation of Cu/O_2_ species. Our work highlights the importance of a mechanistic understanding to improve oxidation processes as well as underlines the use of metal-directed transformations to study the mechanisms by which metalloenzymes functionalize organic molecules.

## Graphical abstract

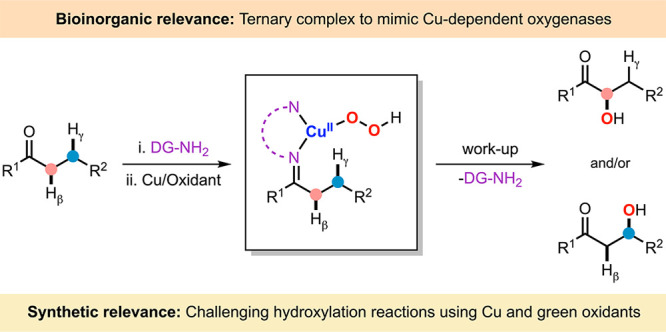

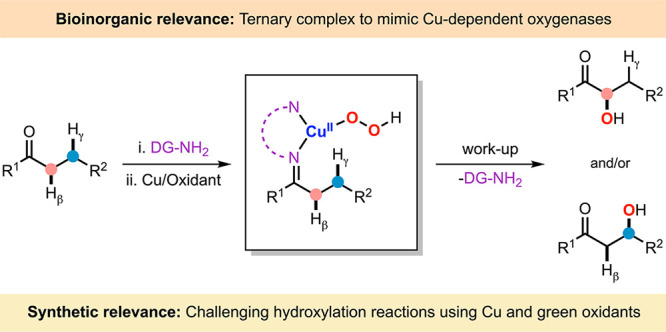

## Key References

-

Garcia-Bosch, I. ; Siegler, M. A. Copper-Catalyzed Oxidation of Alkanes with H_2_O_2_ under a Fenton-like Regime. Angew. Chem., Int. Ed. 2016, 55 (41), 12873–12876 10.1002/anie.20160721627610603.^1^ Copper complexes catalyze the hydroxylation of strong C–H bonds using H_2_O_2_ via formation of O-centered radicals.
-

Trammell, R. ; See, Y. Y. ; Herrmann, A. T. ; Xie, N. ; Díaz, D. E. ; Siegler, M. A. ; Baran, P. S. ; Garcia-Bosch, I. Decoding the Mechanism of Intramolecular Cu-Directed Hydroxylation of sp^3^ C–H Bonds. J. Org. Chem. 2017, 82 (15), 7887–7904 28654755 10.1021/acs.joc.7b01069PMC5792191.^2^ Mechanistic studies on the Cu-directed C–H hydroxylation reactions developed by Schönecker revealed that the oxidations involved the formation of mononuclear species derived from Cu and H_2_O_2_.
-

Zhang, S. ; Goswami, S. ; Schulz, K. H. G. ; Gill, K. ; Yin, X. ; Hwang, J. ; Wiese, J. ; Jaffer, I. ; Gil, R. R. ; Garcia-Bosch, I. Regioselective Hydroxylation of Unsymmetrical Ketones Using Cu, H_2_O_2_, and Imine Directing Groups via Formation of an Electrophilic Cupric Hydroperoxide Core. J. Org. Chem. 2024, 89 (4), 2622–2636 38324058 10.1021/acs.joc.3c02647PMC10877615.^3^ This study revealed that the formation of electrophilic copper­(II) hydroperoxide intermediates leads to the regioselective C–H hydroxylation of unsymmetrical ketones.
-

Petrillo, A. ; Kirchgeßner-Prado, K. F. ; Hiller, D. ; Eisenlohr, K. A. ; Rubin, G. ; Würtele, C. ; Goldberg, R. ; Schatz, D. ; Holthausen, M. C. ; Garcia-Bosch, I. ;  Expanding the Clip-and-Cleave Concept: Approaching Enantioselective C–H Hydroxylations by Copper Imine Complexes Using O_2_ and H_2_O_2_ as Oxidants. J. Am. Chem. Soc. 2024, 146 (37), 25689–25700 39240225 10.1021/jacs.4c07777PMC11823439.^4^ This work reports the first example of an enantioselective C–H hydroxylation reaction using Cu, green oxidants (O_2_ and H_2_O_2_), and chiral directing groups.

## Introduction

Cu-dependent oxygenases and oxidases perform the selective oxidation of organic substrates, including dehydrogenation and hydroxylation transformations, under mild conditions (room temperature, atmospheric pressure) using natural oxidants (O_2_ and/or H_2_O_2_).^5–11^ These Cu-dependent metalloenzymes are involved in the synthesis of important hormones and neurotransmitters (e.g., dopamine hydroxylation to norepinephrine in dopamine β-monooxygenase, DβM^9,12^ ), cellulose degradation (lytic polysaccharide monooxygenases, LPMOs^13–15^ ), small molecule activation (e.g., the oxidation of CH_4_ to CH_3_OH in particulate methane monooxygenase, pMMO^16–18^ ), among other challenging transformations (see Figure 1).^19–21^ Differences in the active centers of these enzymes (ligand identity, number of Cu ions) lead to a broad range of Cu_*n*_/O_2_ intermediates with diverse reactivity.^7,22,23^ For most of them, the reaction mechanism(s) that leads to substrate oxidation is not well understood. For example, several potential Cu active sites have been proposed for pMMO, including mononuclear and dinuclear centers (Figure 1, top left).^11,24–26^ Additionally, the reaction mechanism by which CH_4_ oxidation is coupled with O_2_ reduction is completely unknown (Is O_2_ reduced to H_2_O_2_ before C–H oxidation?^17^ What Cu/O_2_ intermediates are formed? ^27,28^ ). The recent discovery of Cu-dependent metalloenzymes capable of performing novel transformations (e.g., peptide macrocyclization in BURP domain peptide cyclases, BpCs^29^) and new groundbreaking evidence on the reactivity of well-studied enzymes (e.g., Do LPMOs use O_2_ or H_2_O_2_ to oxidize C–H bonds? ^30,31^ Are Cu_2_O_2_ species responsible for C–H hydroxylation in DβM? ^32,33^ ) requires novel approaches to identify the reactive Cu/O_2_ intermediates formed in the enzymes and to understand the reaction mechanism(s) that leads to selective C–H oxidation.

**Figure 1:**
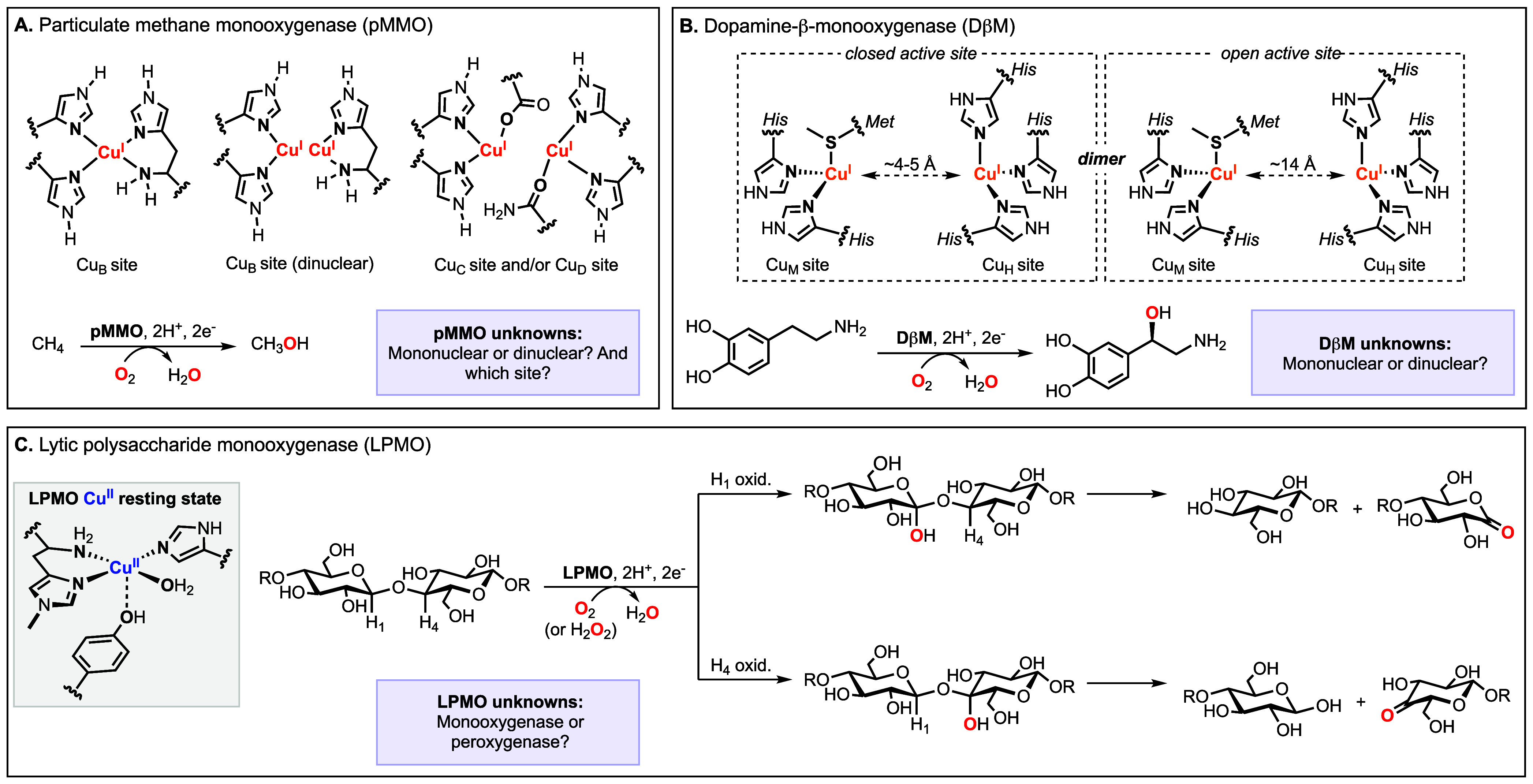
Selected examples of Cu-dependent monooxygenase/peroxygenase enzymes that catalyze C–H hydroxylation, including particulate methane monooxygenase (pMMO, A), dopamine β-monooxygenase (DβM, B), and lytic polysaccharide monooxygenase (LPMO, C).

Inorganic chemists have explored the use of low-weight Cu complexes that can mimic structure, spectroscopy, and/or reactivity of Cu-dependent enzymes.^7,22,34,35^ The main advantage of these systems is that they can be studied in organic solvents (an environment that mimics the hydrophobic nature of the active site of metalloenzymes), which allows for trapping and characterizing reaction intermediates at cryogenic temperatures (e.g., Cu/O_2_ species^34,35^ ). The recent reports on Cu metalloenzymes able to oxidize strong C–H bonds (LPMO and pMMO) have inspired the development of synthetic Cu_*n*_/O_2_ species able to carry out these transformations. Stack found that a L_2_Cu^III^_2_(O^2–^)_2_ core bearing bidentate histamine ligands (similar to the His_brace_ found in pMMO’s Cu_B_ site) promotes the intermolecular oxidation of weak C–H bonds.^8,36^ Tolman has studied the oxidation of C–H bonds using a mononuclear LCu^III^–OH complex, a putative intermediate for LPMO.^37,38^ However, in both cases, the Cu/O_2_ species acted as 1e^–^ oxidants and were not able to carry out the formation of the C–O bond. In 2016, our research lab developed a practical protocol to analyze the catalytic performance of a series of Cu complexes in the oxidation of organic substrates containing strong C–H bonds (e.g., cyclohexane) using H_2_O_2_ as the oxidant (see Figure 2A). The copper complexes derived from tris­((2-pyridyl)­methyl)­amine (TMPA) were among the most active, leading to unprecedented turnover numbers (∼50 TON using 1 mol % of Cu). Alkyl hydroperoxides were the main oxidation products formed, which suggested that these Cu-catalyzed reactions occurred via the formation of nonselective Fenton-like oxidants (hydroxyl and/or hydroperoxyl radicals, see Figure 2B).^1^ Analysis of the products derived from the oxidation of toluene using Cu and H_2_O_2_ provided further evidence of the formation of hydroxyl radicals as active oxidants in these C–H functionalization reactions (Figure 2C). Kinetic experiments led us to propose the reaction mechanism depicted in Figure 2D, in which both Cu^I^ and Cu^II^ are capable of generating the O-centered radicals that lead to the formation of alkyl hydroperoxide products. Early work by Barton and co-workers showed that combining Cu salts and H_2_O_2_ in pyridine can promote the hydroxylation of strong C–H bonds, but those reactions were carried out under excess equivalents of the C–H substrate (H_2_O_2_ as limiting reagent), hence precluding the generation of oxidation products in high yields.^39,40^

**Figure 2:**
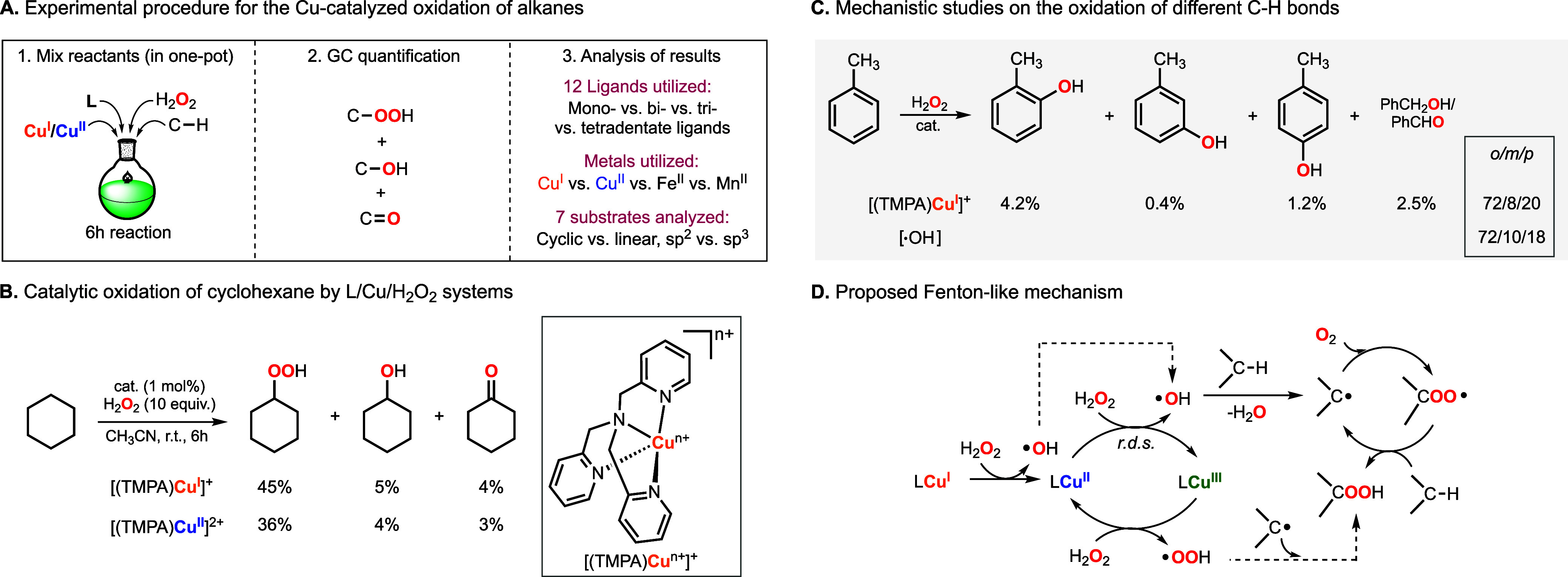
Cu-catalyzed nonselective oxidation of strong C–H bonds using H_2_O_2_ as oxidant. (A) Experimental procedure for the generation of the Cu complexes that will catalyze the oxidation of C–H substrates with H_2_O_2_. (B) Results obtained in the oxidation of cyclohexane using Cu-TMPA complexes and H_2_O_2_. (C) Oxidation of toluene using the Cu/TMPA/H_2_O_2_ system that leads to a distribution of products similar to the ones observed for the hydroxyl radical. (D) Proposed mechanism for the Cu-catalyzed oxidation of alkanes with H_2_O_2_.

To achieve regioselectivity, Cu-dependent oxygenases bind the organic substrate before exposing the Cu center to the oxidant, a reaction that leads to the formation of a highly organized ternary complex prior to substrate hydroxylation (i.e., metal–substrate–oxidant adduct^41^). During the last decades, several research groups have reported that mononuclear and dinuclear Cu/O_2_ species promote intramolecular C–H hydroxylation reactions resembling the reactivity observed in enzymes (Figure 3A).^42–47^ The so-called substrate-binding ligand approach^45^ consists of exposing Cu complexes (Cu^I^ or Cu^II^) to naturally relevant oxidants (O_2_ or H_2_O_2_) to produce metastable Cu/O_2_ species that decay to oxidize the ligand scaffold (see also relevant references by the Reinaud^48,49^ and Rogić^50^ groups on Cu-mediated oxidations using a similar strategy).

**Figure 3:**
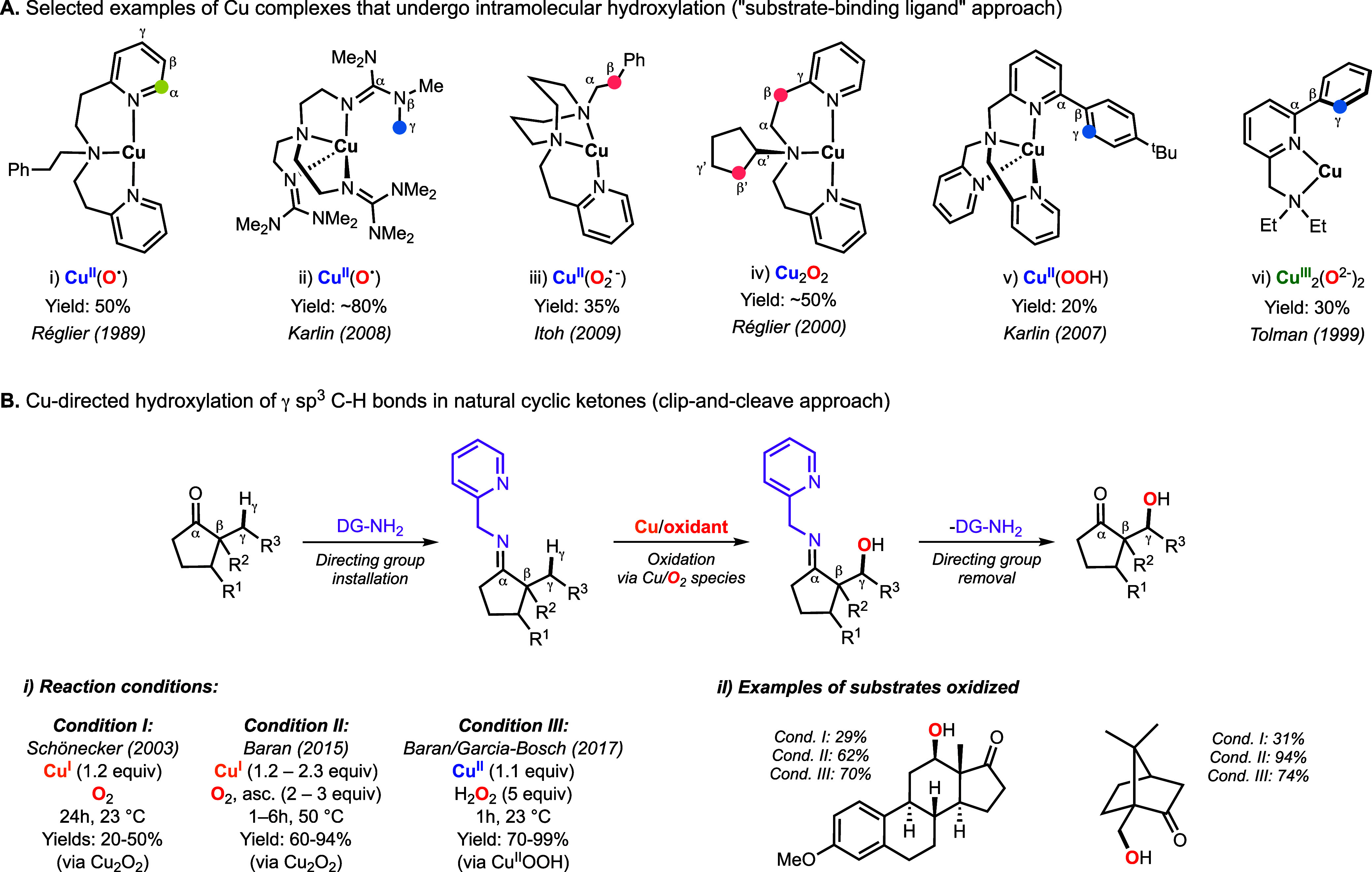
Cu-promoted intramolecular oxidation reactions observed in the substrate-binding ligand approach (A) and click-and-cleave approach (B). Note: according to Schönecker, the positions adjacent to the N coordinated to the Cu are named α, and the subsequent positions are named β, γ, δ, etc. This might lead to confusion because in organic chemistry, the α position of a carbonyl group is C_β_ in Schönecker’s nomenclature. As shown in Figure 3A, Schönecker naming is useful because some Cu systems perform C_α_ functionalization. Note: Schönecker first^51^ and Baran later^52^ proposed that C–H hydroxylation occurred via formation of dinuclear species (Cu_2_O_2_). In our study, we proposed that the “so-called” Schönecker–Baran oxidations occur via formation of mononuclear species (Cu^II^OOH).^2^

Inspired by the ability of Cu/O_2_ to perform intramolecular oxidations, Schönecker and co-workers reported the first example of a Cu-directed C–H hydroxylation reaction (Figure 3B).^51^ The authors utilized 2-picolylamine as a directing group, stoichiometric amounts of Cu^I^, and dioxygen as the oxidant to carry out the regioselective γ-C–H hydroxylation of natural cyclic ketones, including steroids and (*R*)-camphor (Note: to be consistent with the nomenclature used by Schönecker and co-workers, the C atoms adjacent to the N coordinated to the Cu ion are named C_α_. We realize that this is somewhat confusing in the context of functionalization of ketones and aldehydes because the α positions are commonly the ones adjacent to the C atom of the carbonyl group. Using Schönecker’s nomenclature, the installation of the DG produces an imine with a N atom that will be coordinated by Cu and with an adjacent C atom that will be named C_α_ (C derived from the carbonyl), making the C atoms adjacent to C_α_, C_β_, which would commonly be α position of the carbonyl group). In 2015, Baran and co-workers improved the Schönecker oxidation by using Cu in excess (up to 2.3 equiv) and by combining O_2_ with stoichiometric amounts of reducing agents (up to 2.3 equiv), which led to a substantial increase in the reaction yields.^52^ Based on the yields achieved (below 50% in the absence of reductant), both research groups proposed the formation of dinuclear Cu/O_2_ species (Cu^II^_2_(O_2_^2–^) and/or Cu^III^_2_(O^2–^)_2_) as hydroxylating reagents because these are known to act as 2e^–^ oxidants (i.e., after their formation, only half equivalent of the substrate–ligand is oxidized).^7,34^ However, no spectroscopic evidence of the Cu species formed during the oxidation reactions was provided. In 2017, our research lab collaborated with the Baran group to determine the mechanism by which the Schönecker oxidations occur.^2^ Our data suggested that the “real” oxidant in these oxidations was hydrogen peroxide (derived from the 2H^+^/2e^–^ reduction of O_2_ and solvent oxidation) and that mononuclear Cu/O_2_ species were involved in C–H hydroxylation. Based on the mechanistic analysis, we developed reaction conditions that utilized H_2_O_2_, leading to higher yields, lower costs (use of Cu^II^ without reductant), shorter reaction times, and improved practicability (i.e., no use of an O_2_ balloon and reactions carried out at room temperature).

## Cu-Directed C–H Hydroxylation Reactions: Discovery, Scope, and Mechanism

Our interest in Cu-directed oxidations is 2-fold. On one hand, this approach allows for developing synthetic protocols to perform selective C–H hydroxylation reactions in a cheap, safe, practical, and selective manner. On the other hand, we can also gather mechanistic understanding of the reaction pathways by which Cu-dependent metalloenzymes catalyze the oxidation of organic substrates (Figure 4A). Our work on this topic typically includes the synthesis and characterization of the Cu complexes derived from the substrate–ligand systems, oxidation of these complexes under varying conditions (using O_2_ or H_2_O_2_ as oxidants at different temperatures and with different solvents), characterization of the Cu/O_2_ intermediates formed during the oxidation, and other mechanistic experiments (e.g., use of radical traps to intercept C-centered radical intermediates). As we have stated, our study on the mechanism of the so-called Schönecker–Baran oxidations revealed that the C–H hydroxylation reaction occurred via the formation of mononuclear Cu^II^OOH species, which allowed for developing reaction conditions based on the use of Cu and H_2_O_2_.^2^ In 2019, we expanded the substrate scope of the Cu-directed C–H hydroxylation reactions (Figure 4B).^53^ The oxidations were carried out in acetone using 2-picolylamine as the directing group, 1 equiv of Cu^I^, and 5 equiv of H_2_O_2_ (30% in H_2_O), which were able to perform the γ-hydroxylation of sp^2^ C–H bonds (e.g., hydroxylation of substituted benzophenones), the β-hydroxylation of sp^3^ C–H bonds (e.g., hydroxylation of 2-adamantone), and the γ-hydroxylation of sp^3^ C–H bonds (e.g., hydroxylation of (*R*)-camphor).

**Figure 4:**
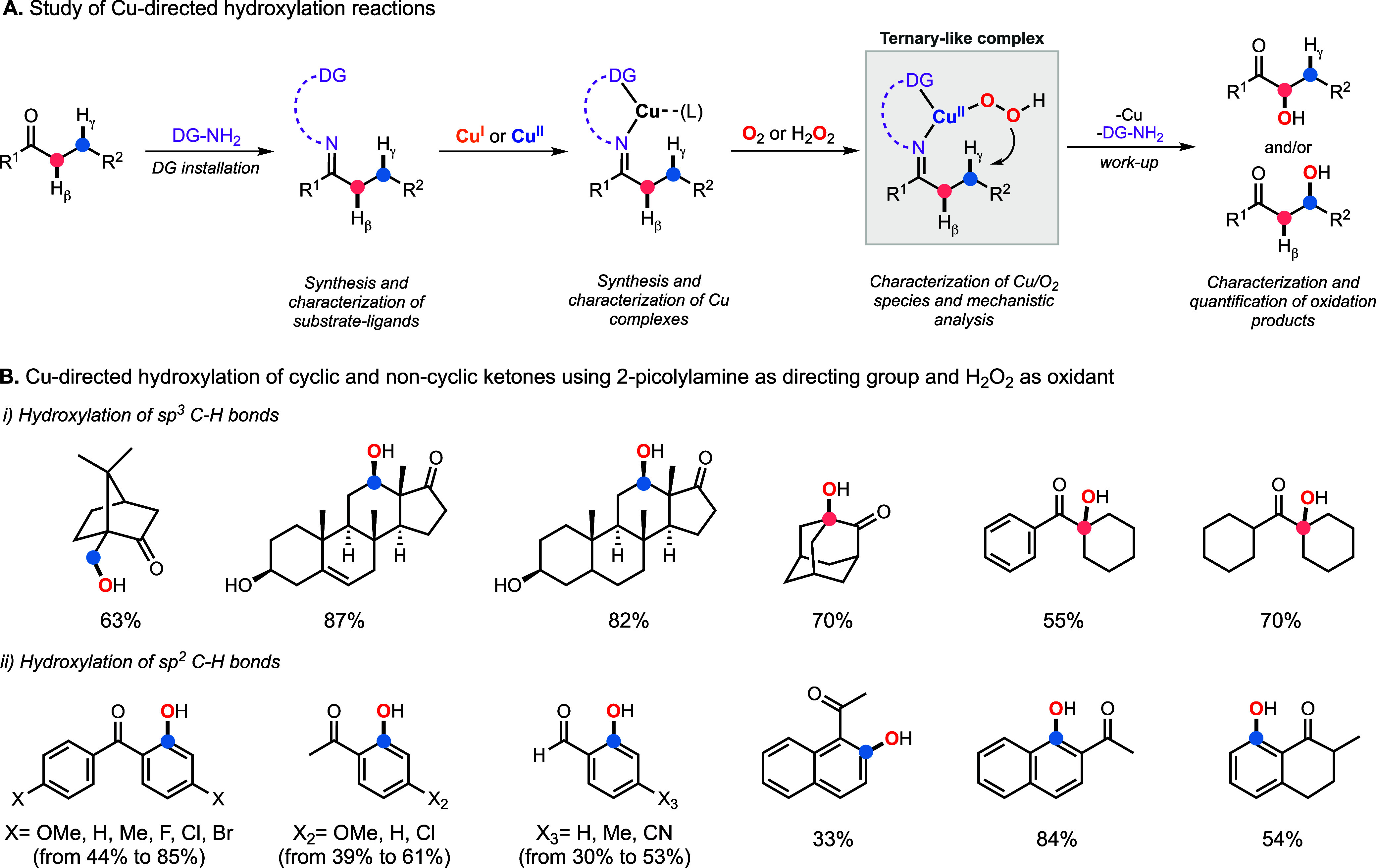
Approach followed in our work on Cu-directed C–H hydroxylation reactions (A) and some examples of the hydroxylation products synthesized (B).

The mechanism by which Cu promotes C–H bond oxidation is shown in Figure 5A.^2,53^ Our mechanistic studies suggest that upon coordination to the substrate–ligand (species A in Figure 5A), Cu^I^ reacts with dioxygen to produce a metastable Cu^II^-superoxide species, which decomposes to produce the corresponding Cu^II^ complex and superoxide anion in solution (from species B to C in Figure 5A). The superoxide is converted to H_2_O_2_ via solvent oxidation (note: the products derived from acetone oxidation were characterized and quantified), which then reacts with the Cu^II^ substrate–ligand complex to produce a Cu^II^OOH intermediate (species D in Figure 5A). The Cu^II^OOH species was generated in a quantitative fashion via the addition of H_2_O_2_ to the independently synthesized Cu^I^ and Cu^II^ substrate–ligand complexes, which allowed its characterization by UV–vis and EPR spectroscopy. Kinetic analysis of the reactions suggested that the Cu^II^OOH intermediate accumulated before the rate-determining step of the reaction. The mechanism by which the Cu^II^OOH undergoes O–O bond cleavage and C–H hydroxylation occurs was proposed to be dependent on the substrate oxidized (see Figure 5B). In the γ-hydroxylation of sp^3^ C–H bonds, the Cu^II^-hydroperoxide intermediate undergoes homolytic O–O bond cleavage to generate a Cu^II^-oxyl species and hydroxyl radical (from species E to species I in Figure 5B). The intramolecular reaction between the OH-radical and the substrate–ligand then produces a C-centered radical, which leads to C–O bond formation by reaction with the Cu^II^-oxyl core (from species J to species E in Figure 5B). Both hydroxyl and C-centered radicals were trapped using external C–H substrates and halogenated solvents, respectively.^2^ In the γ-hydroxylation of sp^2^ C–H bonds, we proposed that the Cu^II^-hydroperoxide oxidizes the substrate in a concerted fashion in which the O–O bond cleavage and C–O bond formation occur in one step (in Figure 5B, from species D to species E′ via formation of species K and species L). Our mechanistic proposal was supported by kinetic isotope measurements (an inverse KIE was measured, consistent with the C-sp^2^ to C-sp^3^ change in hybridization during the r.d.s.) and by DFT computations.^53^

**Figure 5:**
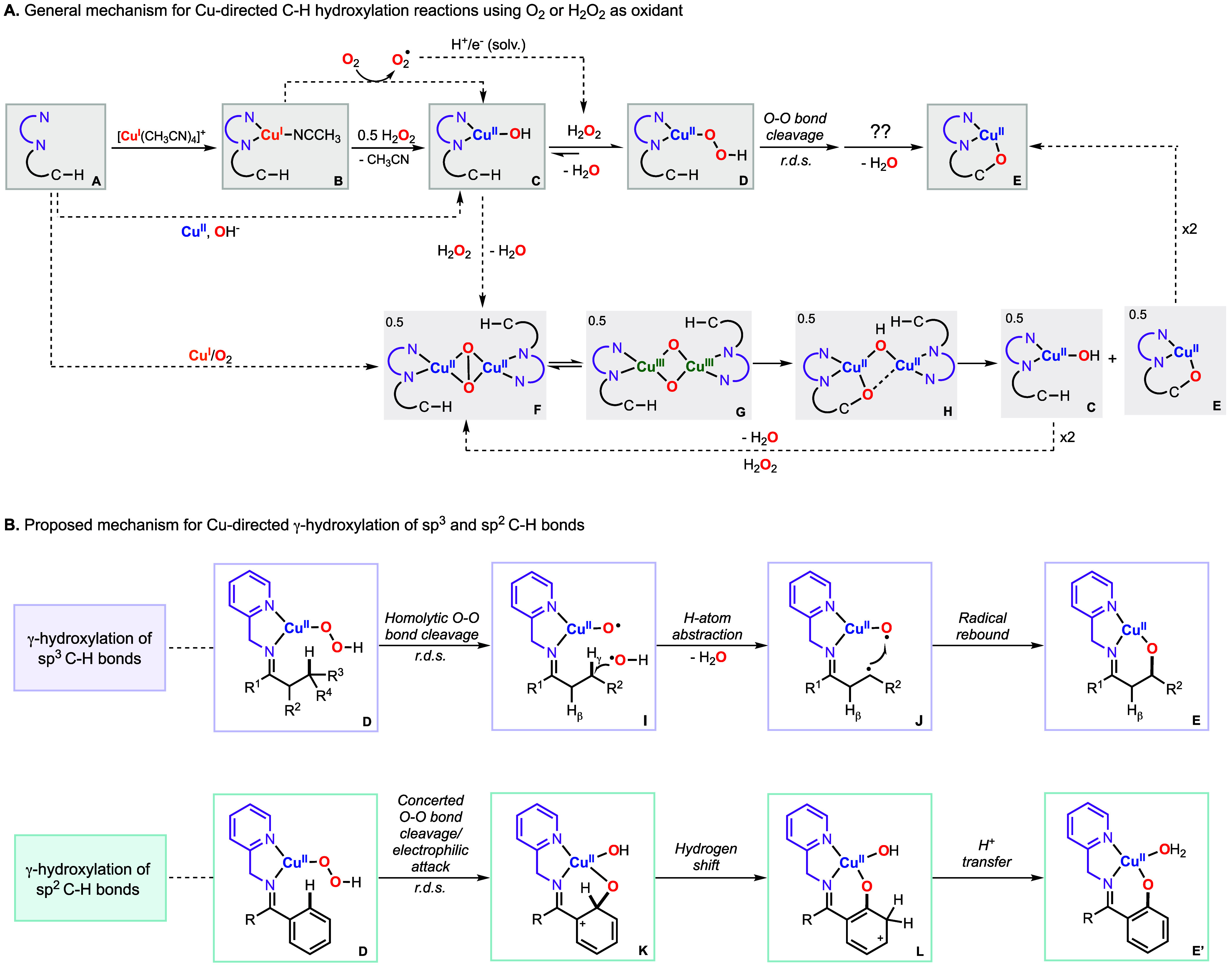
(A) General mechanism for intramolecular C–H hydroxylation via the formation of mononuclear or dinuclear Cu/O_2_ species (A). Proposed reaction mechanisms for the intramolecular γ-hydroxylation of sp^3^ and sp^2^ C–H bonds (B).

While our data suggest that mononuclear Cu^II^OOH species are responsible for C–H hydroxylation, the involvement of dinuclear Cu_2_O_2_ cores should not be ruled out (see species F, G, and H in Figure 5A). Other research groups have found that Cu complexes bearing bidentate ligands that produce dinuclear Cu/O_2_ species (by reacting Cu^I^ with O_2_ or Cu^II^ with H_2_O_2_) can also perform intramolecular C–H hydroxylation reactions.^54,55^ In 2021, we studied the ability of bi-, tri-, and tetradentate ligands to perform Cu-directed hydroxylation reactions, and we found that higher yields were achieved when bidentate directing groups (rather than tri- and tetradentate) and H_2_O_2_ (rather than O_2_) were used.^56^ Interestingly, some of the substrate–ligands utilized led to the formation of dinuclear Cu/O_2_ species (Cu^II^_2_(O_2_^2–^) and Cu^III^_2_(O^2–^)_2_) that did not lead to intramolecular C–H hydroxylation, reinforcing the idea that mononuclear species might be involved in substrate oxidation. A recent study by Murakami and co-workers described a new class of Cu-dependent metalloenzymes that cleave cellulose in an unprecedented fashion (not an LPMO enzyme) following an exo-type mechanism to generate cellobionic acid.^57^ The authors proposed that this enzyme adopts a homodimer configuration in which a mononuclear Cu active center reduces O_2_ to H_2_O_2_ (oxidase reactivity) and the other mononuclear center uses the H_2_O_2_ to carry out the hydroxylation of the substrate (peroxygenase chemistry), a reactivity that resembles the mechanism in Cu-directed γ-hydroxylation of C–H bonds using 2-picolylamine as directing group.

## Regioselectivity in Cu-Directed C–H Hydroxylation Reactions

In 2024, we analyzed the Cu-directed hydroxylation of unsymmetrical ketones using 2-picolylamine as directing group and H_2_O_2_ as oxidant (see Figure 6).^3^ Our study was primarily focused on the hydroxylation of unsymmetrical benzophenones, although other unsymmetrical ketones were also analyzed. When 2-substituted and 4-substituted benzophenones are used as substrates, the installation of the directing group can produce two imine isomers (*E* and *Z*), which can generate two oxidation products derived from γ-sp^2^ C–H hydroxylation. When 2-substituted benzophenones were used, we observed the formation of one of the imine isomers (*Z* isomer, with the DG pointing toward the unsubstituted phenyl ring) that upon addition of Cu^I^ and H_2_O_2_ only produced the corresponding *Z*-hydroxylation product. Conversely, the use of 4-substituted benzophenones led to the formation of the 2 imine isomers (*E* and *Z*) that were oxidized to the corresponding hydroxylation products (*E* and Z). Strikingly, the ratio of the substrate–ligand imine isomers (*E*/*Z* ∼ 60/40) was different from the ratio of hydroxylation products, favoring the oxidation of the phenyl rings containing electron-donating substituents. For example, installation of 2-picolylamine to 4-bromobenzophenone produced the two imine isomers with an *E*/*Z* ratio of 62/38 (in the *E* isomer the DG is pointing toward the ring with Br), that upon oxidation produce the hydroxylation products in a ratio of 30/70 (the hydroxylation occurred mainly in the unsubstituted ring). The regioselectivity was enhanced by using 4,4′-disubstituted benzophenones such as 4-methoxy-4′-trifluorobenzophenone, whose Cu-directed hydroxylation favored the oxidation of the phenyl ring containing the MeO substituent (ratio of 86/14). For 4-substituted and 4,4′-disubstituted benzophenones, our mechanistic studies suggested the existence of fast isomerization equilibria between the imine isomers before the rate-determining step of the reaction, in which an electrophilic mononuclear Cu^II^OOH favored the hydroxylation of the electron-rich arene ring (Figure 6B). Conversely, we proposed that for 2-substituted benzophenones, slow (or no) equilibria between the imine isomers occurred, leading to hydroxylation of the unsubstituted arene ring.

**Figure 6:**
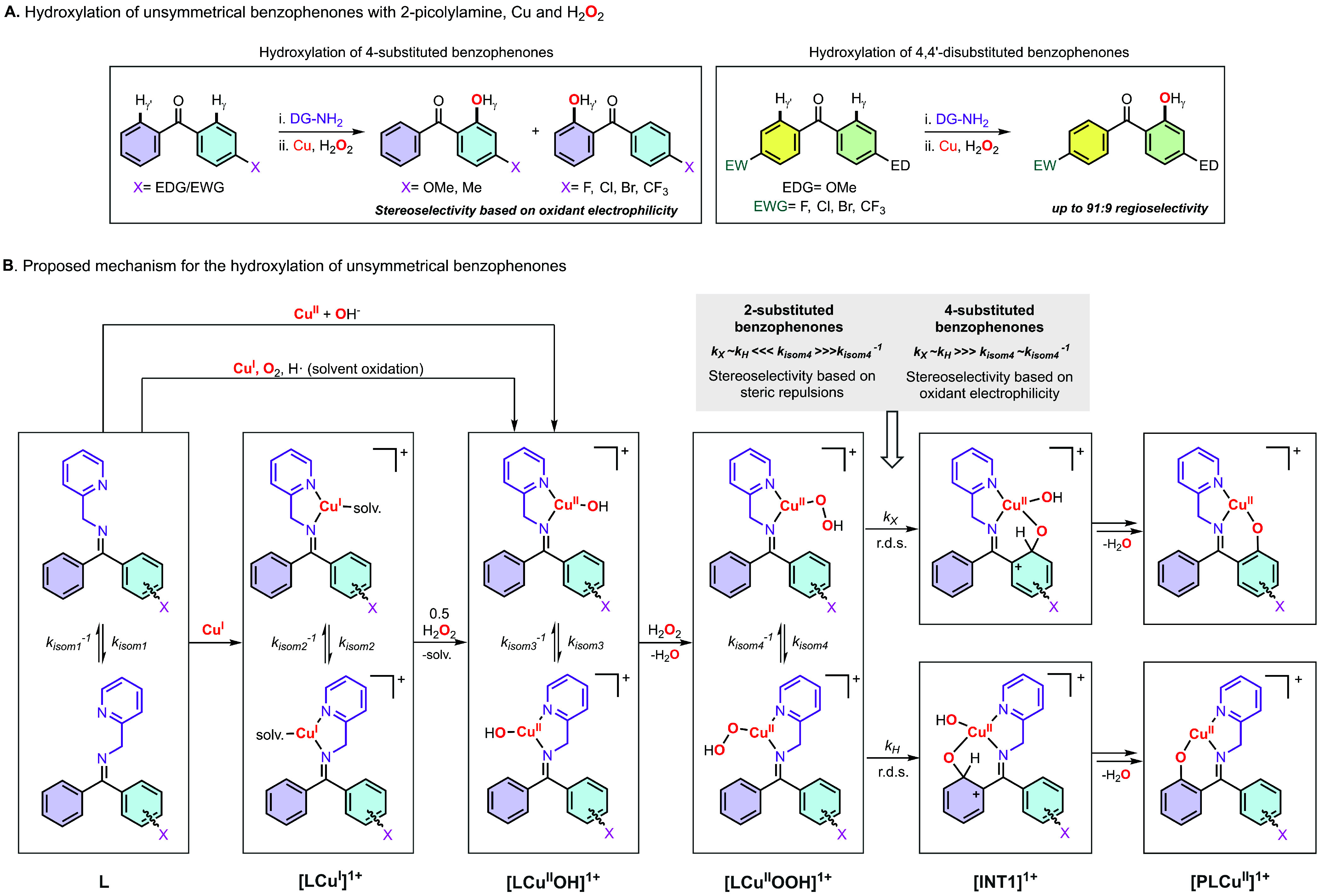
Scope (A) and mechanism (B) of the Cu-directed hydroxylation of unsymmetrical benzophenones using 2-picolylamine as the directing group.

The regioselectivity in Cu-directed C–H hydroxylation reactions using 2-picolylamine and H_2_O_2_ is based on three factors (Figure 7). The first factor to consider is the formation of 1 or 2 imine substrate–ligands. For example, the installation of 2-picolylamine to 2-methyl-1-tetralone forms only the imine isomer in which the directing group is pointing toward the arene ring, which allowed for carrying out the γ-sp^2^ C–H hydroxylation in a selective fashion.^53^ Conversely, Schöenebeck and co-workers have shown that 2-methyl-1-tetralone can undergo β-sp^3^ C–H hydroxylation using Cu_2_O, a strong base, and O_2_ as oxidant (Figure 7A).^58^ Interestingly, the formation of a sole imine isomer can also lead to the regioselective hydroxylation of sp^2^ C–H bonds of unsymmetrical ketones containing unsymmetrical arene substituents. For example, we observed that the Cu-directed hydroxylation of 2-acetonaphthone led to the selective hydroxylation of the position 1 of the naphthyl ring (84% yield) leaving the position 3 (also a γ-sp^2^ C–H bond) unreacted (Figure 7B).^53^ Thus, the regioselectivity of the Cu-directed hydroxylation of 2-methyl-1-tetralone and 2-acetonaphthone seems to be dictated by the substrate–ligand topology. In contrast, the regioselectivity in the Cu-directed hydroxylation of unsymmetrical 4-substituted benzophenones relies on the electrophilicity of the Cu/O_2_ species formed, which favors the hydroxylation of electron-rich arene rings (see Figure 7C).^3^

**Figure 7:**
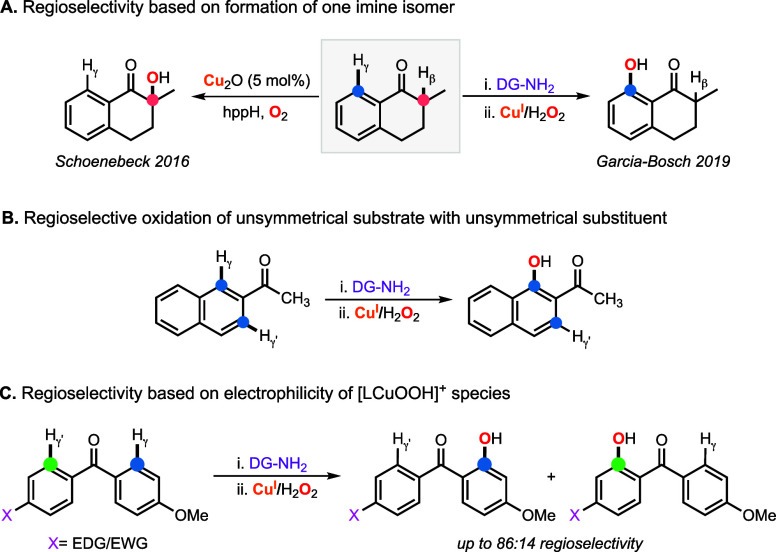
Regioselective oxidation of substrate–ligands with multiple C–H bonds using 2-picolylamine as directing group based on the formation of one imine isomer (A), based on the functionalization of a substrate with unsymmetrical substituents (B), and based on the formation of an electrophilic oxidant (C).

In 2024, we described the Cu-directed hydroxylation of benzophenones using 2-(2-aminoethyl)­pyridine as directing group (Figure 8).^59^ To our surprise, we observed that the substrate–ligands derived from 4-methoxy, 4-fluoro, and 4-phenylbenzophenone led to the formation of an additional oxidation product attributed to β-ipso oxidation (for 4-methoxy-benzophenone, the ipso-oxidation was highly favored). As shown in Figure 8, ipso-oxidation leads to the formation of a C_β_–O bond that triggers an unusual 1,2-rearrangement to produce a C_α_–C_γ_ bond (with apparent shift of the substituent from the 4 to the 5 position of the phenyl ring). Our mechanistic studies suggested that after generation of a mononuclear Cu^II^OOH species, this intermediate undergoes a concerted O–O bond cleavage with concomitant electrophilic attack to the C_γ_ of the phenyl ring to produce the γ-C–H hydroxylation product, a reaction pathway analogous to the one proposed in the γ-sp^2^ C–H hydroxylation reactions in the systems with 2-picolylamine. However, the use of 2-(2-aminoethyl)­pyridine can also trigger β-ipso oxidation. Computations suggested that this unusual oxidation occurs in a stepwise fashion in which the O–O bond cleavage leads to the formation of a Cu^III^-oxyl-hydroxo intermediate that performs the electrophilic ipso-oxidation of the aryl ring. The 1,2-rearrangement was hypothesized to occur due to the strain release upon conversion of the spiro intermediate formed after the ipso-attack (from a 5-membered ring with Cu–N_im_–C_α_–C_β_–O to a 6-membered ring with Cu–N_im_–C_α_–C_γ_–C_β_–O).

**Figure 8:**
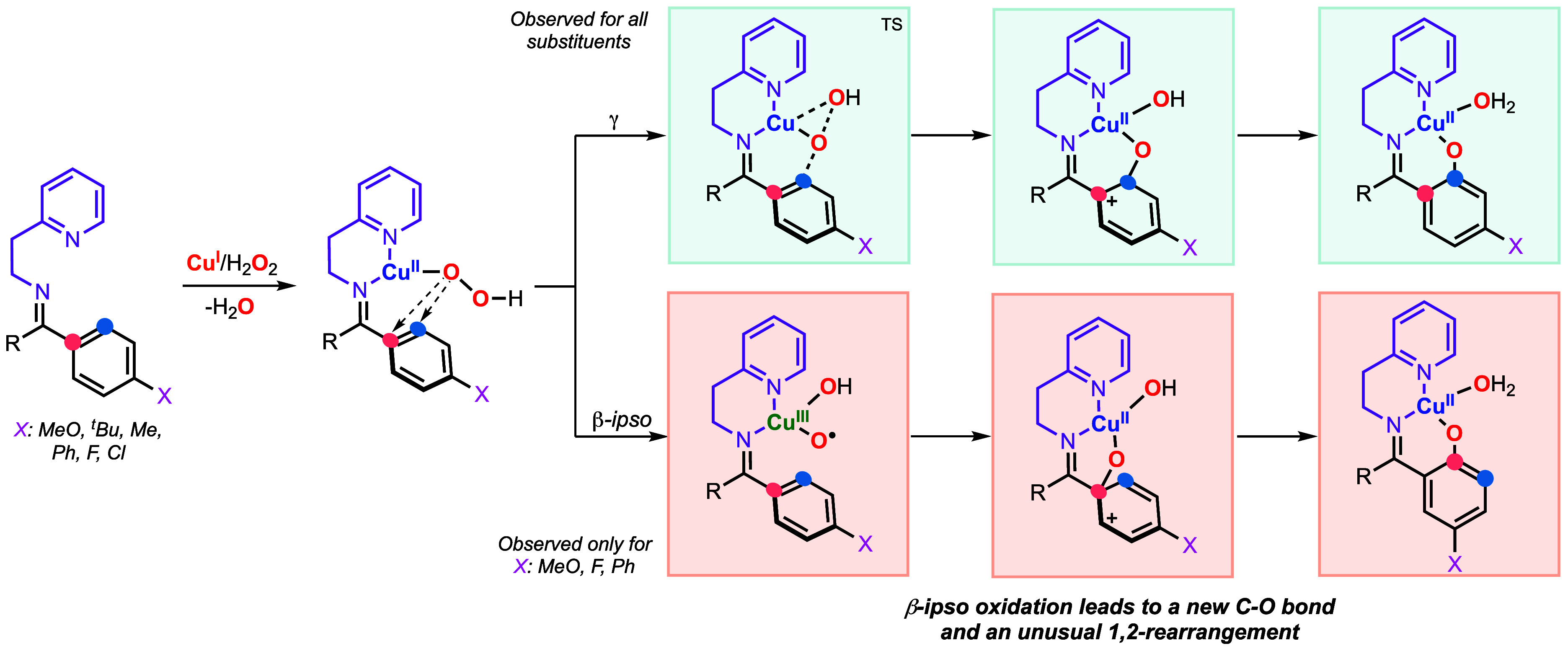
Proposed mechanisms for the *ipso*-oxidation and γ-hydroxylation of unsymmetrical benzophenones using Cu, H_2_O_2_, and 2-(2-aminoethyl)­pyridine as directing group.

The installation of 2-(2-aminoethyl)­pyridine to unsymmetrical benzophenone also produced two imine isomers. When compared to the oxidation of the systems derived from 2-picolylamine (which only led to the 2 products derived from γ-sp^2^ C–H hydroxylation), the systems derived from 2-(2-aminoethyl)­pyridine can potentially form four oxidation products derived from β- and γ-sp^2^ oxidation (see Figure 9). Analysis of the oxidation of unsymmetrical disubstituted benzophenones suggested that the Cu/O_2_ oxidant formed in the 2-(2-aminoethyl)­pyridine systems is more selective than the one formed in the 2-picolylamine analogues. For example, the oxidation of 4-methoxy-4′-methylbenzophenone using 2-picolylamine slightly favored the oxidation of the arene containing the MeO substituent (53/47 ratio), while 2-(2-aminoethyl)­pyridine had an enhanced preference (81/19 ratio). For all of the systems derived from 4-methoxy-4′-X-benzophenones and 2-(2-aminoethyl)­pyridine, we observed a high preference for the β-oxidation of the arene ring containing the MeO substituent.

**Figure 9:**
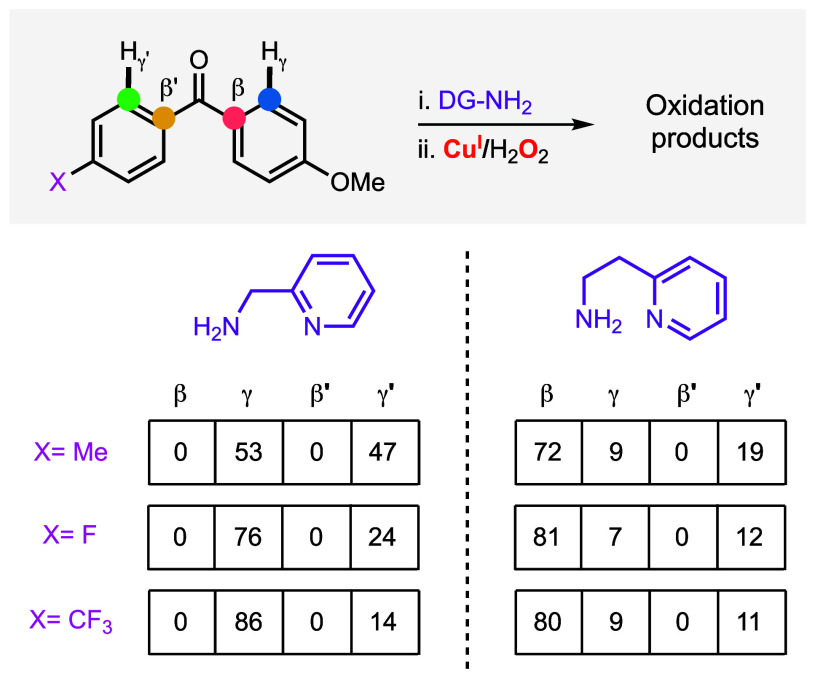
Comparison of the regioselectivity observed in the Cu-directed hydroxylation of unsymmetrical benzophenones using 2-picolylamine or 2-(2-aminoethyl)­pyridine as directing groups.

## Enantioselectivity in Cu-Directed C–H Hydroxylation Reactions

In collaboration with the Holthausen group and the Schindler group, we recently reported the first example of enantioselective Cu-directed C–H hydroxylation reactions (see Figure 10).^4^ The use of chiral bidentate directing groups such as (1*R*, 2*R*)-cyclohexane-1,2-diamine, (1*R*, 2*R*)-*N* ^1^,*N* ^1^-dimethylcyclohexane-1,2-diamine and (1*R*)-1-(2-pyridinyl)­ethanamine resulted in the regio- and enantioselective β-hydroxylation of 2-cyclohexanone and γ-hydroxylation of 1-acetyladamantane. The substrate–ligands were oxidized using three different oxidation conditions that were previously optimized in our laboratories (based on the use of Cu^I^ + O_2_, Cu^I^ + H_2_O_2_, and Cu^II^ + H_2_O_2_) in different organic solvents (Figure 10A). Better yields and enantioselectivities were obtained for the substrate–ligands derived from 1-acetyladamantane. For the substrate–ligand derived from (1*R*, 2*R*)-*N* ^1^,*N* ^1^-dimethylcyclohexane-1,2-diamine and 1-acetyladamantane, we found that C–H hydroxylation occurred only when Cu^I^ and O_2_ were used. Time-resolved UV–vis spectroscopy measurements carried out at cryogenic temperatures revealed that these systems were oxidized by dinuclear Cu^III^_2_(O^2–^)_2_ (Figure 10B). Conversely, the hydroxylation of the substrate–ligands containing pyridine were enhanced when Cu^II^ and H_2_O_2_ were used via the formation of mononuclear Cu^II^OOH intermediates. Our results indicated that multiple Cu/O_2_ species can lead to regio- and enantioselective C–H hydroxylation and that their formation is dependent on the directing group identity (e.g., *N*-alkylic vs pyridinic directing groups) and the oxidation conditions utilized (e.g., Cu^I^/O_2_ vs Cu^II^/H_2_O_2_).

**Figure 10:**
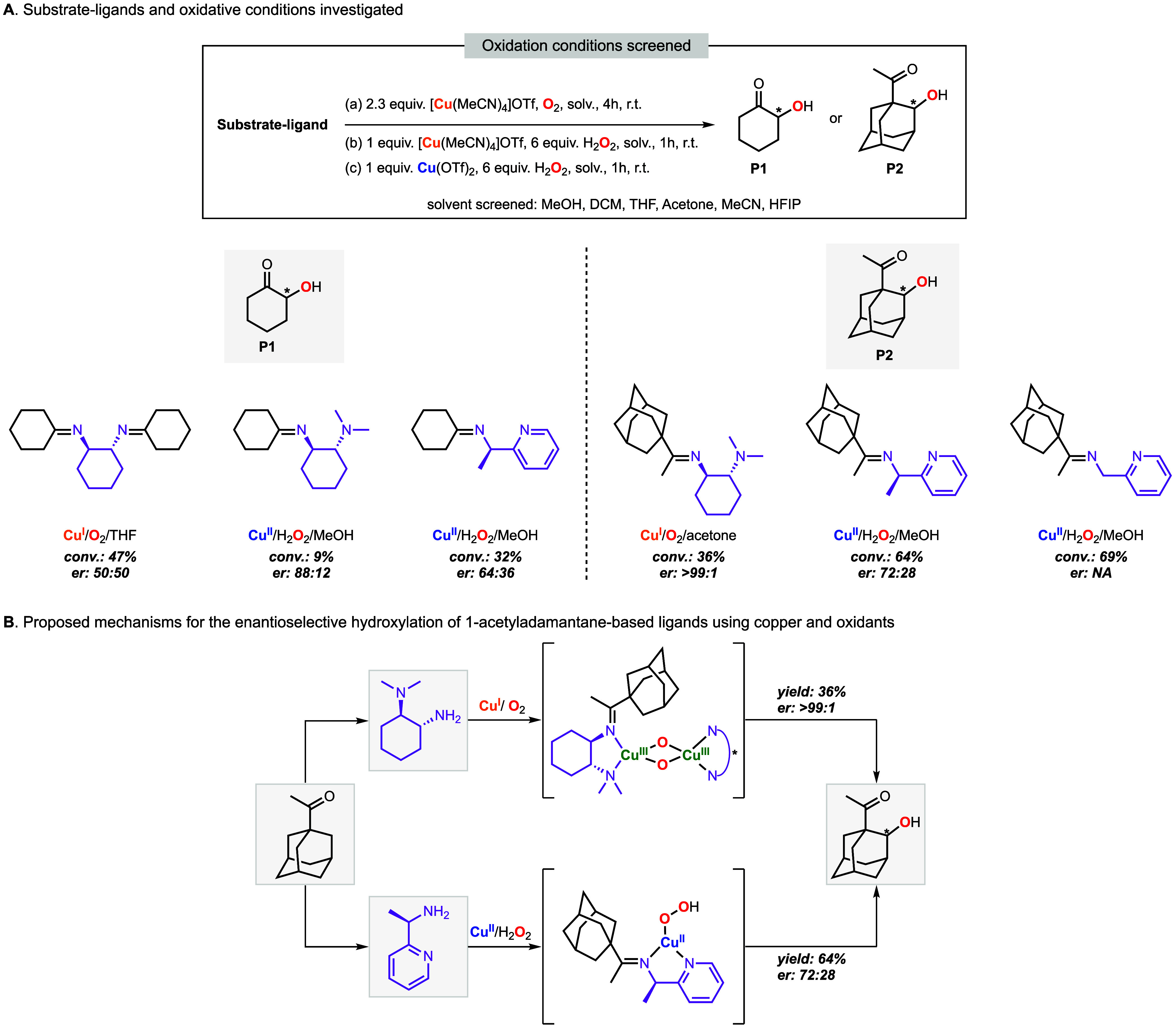
Copper-promoted enantioselective C–H hydroxylation of cyclohexanone-based and 1-acetyladamantane-based substrate–ligands using O_2_ and H_2_O_2_ as oxidants, including the reaction conditions screened (A) and mechanisms proposed (B).

## Concluding Remarks and Future Outlook

As we have shown, work on Cu-directed C–H hydroxylation provides new insights into the reaction mechanism(s) by which Cu-dependent monooxygenases and peroxygenases oxidize organic substrates.^31,60,61^ Mechanistic understanding has led to the development of new oxidation protocols based on the use of Cu and H_2_O_2_, which have been applied in the total synthesis of complex organic molecules via early stage and late-stage C–H functionalization.^62–69^ Despite the many significant advances made during the past decade, the journey has just begun. We believe that the next steps should be focused on increasing the variety of oxidative transformations that can be carried out with this approach. First, we aim to expand the scope of ketone and aldehyde substrates that can be functionalized (e.g., developing protocols to perform the δ-C–H hydroxylation of sp^2^ and sp^3^ substrates in a selective fashion^70^) and carry out mechanistic studies to understand the underpinning principles that govern regioselectivity in these transformations (e.g., Is the β/γ/δ selectivity largely determined by the substrate or can it be controlled via DG modification? Can different Cu/O_2_ species lead to different selectivity?). Second, we hypothesize that we can adapt Cu-directed oxidations to perform the C–H hydroxylation of amine substrates using aldehydes as DGs, a transformation that would produce amino alcohols, which are widespread motifs in natural products and pharmaceuticals.^71–73^

One of the main drawbacks of the directed approach is the use of stoichiometric amounts of DG and metal and the need to install the DG (isolation of the substrate–ligand) before oxidation. Over the past decade, Yu and others have pioneered the use of transient DGs in metal-directed C–H functionalization reactions.^74–77^ This approach (usually used in Pd catalysis and only two examples with Cu^77,78^ ) relies on the reversible formation of an imine bond, which might allow for performing Cu-directed C–H hydroxylation reactions using catalytic amounts of DG and Cu.

Research on the bioinorganic chemistry of Cu is more alive than ever.^23,79,80^ In the past few years, several new Cu-dependent metalloenzymes capable of performing unprecedented organic transformations have been discovered. These include BURP domain peptide cyclases^29^ (BpCs, which catalyze the oxidative macrocyclization of peptides via C–C, C–N, or C–O bond formation between aromatic amino acids and unactivated C–H bonds of other amino acids), Cu-dependent halogenases and pseudohalogenases^81^ (ApnU, which catalyze sp^3^ C–H chlorination, bromination, iodination, and thiocyanation), among others.^82^ We believe that the development of synthetic inorganic complexes (including Cu-directed systems) capable of mimicking the structure, spectroscopy, and reactivity of the active sites of these enzymes will provide insights into the mechanisms by which these natural catalysts perform these oxidations and will lead to the development of useful synthetic protocols to carry out these challenging organic transformations.
